# Supplementary material for: Systems Genetic Identification of Mitochondrion-Associated Alzheimer’s Disease Genes and Implications for Disease Risk Prediction
Source: Biomedicines. 2022 Jul 24;10(8):1782. doi: 10.3390/biomedicines10081782 (PMC9330299; doi:10.3390/biomedicines10081782)
Supplement: Supplementary file 1 [file biomedicines-10-01782-s001.zip › SUPPLEMENT FIGURES-r1.pdf]

SUPPLEMENTARY FIGURES

Systems genetic identification of mitochondrion-associated Alzheimer's disease genes and implications for disease risk prediction

Authors: Xuan Xu; Hui Wang; David A. Bennett; Qing-Ye Zhang; Gang Wang; Hong-Yu Zhang*

Supplement Figure S1. ADNI gene differentially expression analysis
Supplement Figure S2. GO enrichment analysis in key modules
Supplement Figure S3. KEGG enrichment analysis in key modules
Supplement Figure S4. The overall importance ranking of hub genes
Supplement Figure S5. **Transcriptional expression profiles of the top 10 hub genes in the developing human brain across genders**

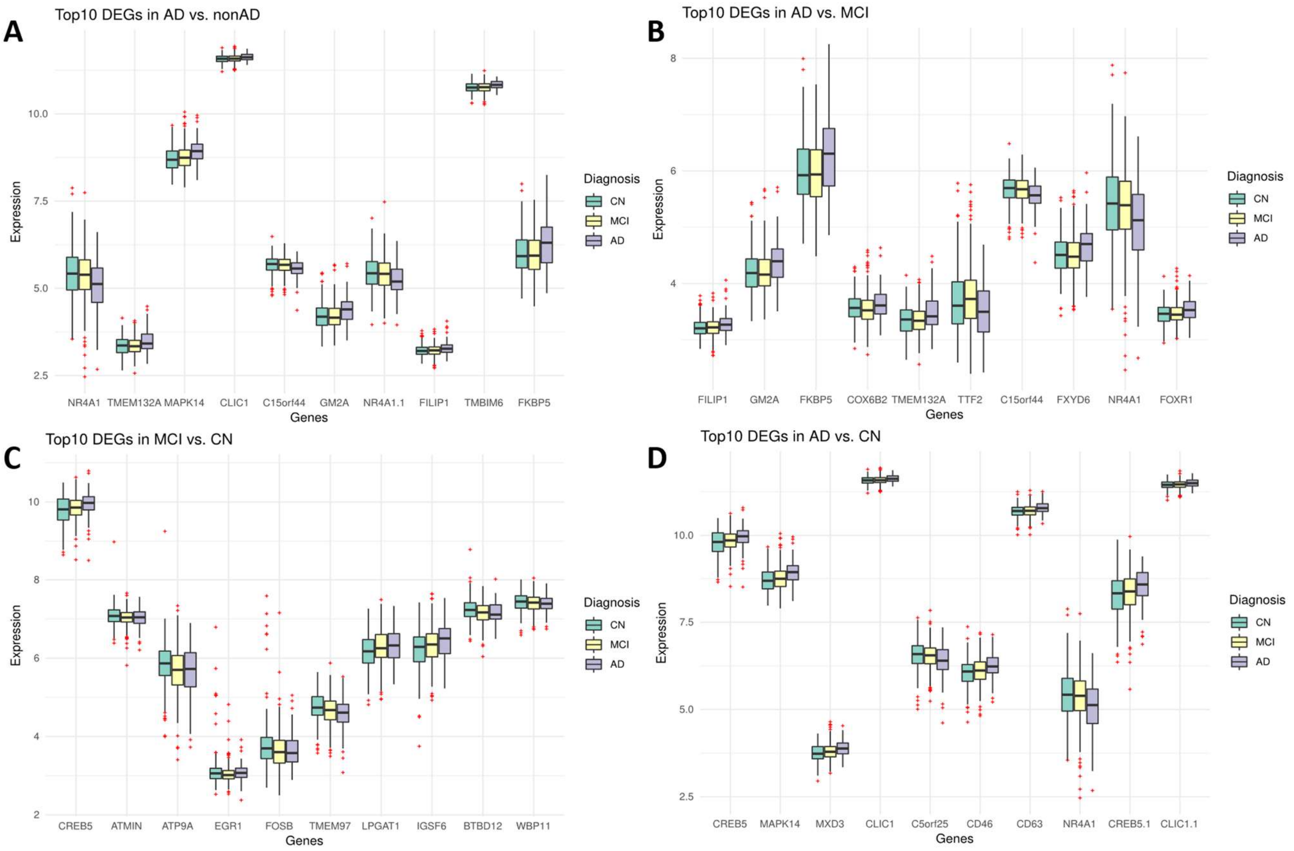

Supplement Figure S1. ADNI gene differentially expression analysis

The top10 differentially expressed genes (DEGs) in four contrast groups. (A) AD vs. no dementia. (B) AD vs. MCI. (C) MCI vs.CN. (D) AD vs.CN (**Table S3 to S6**).

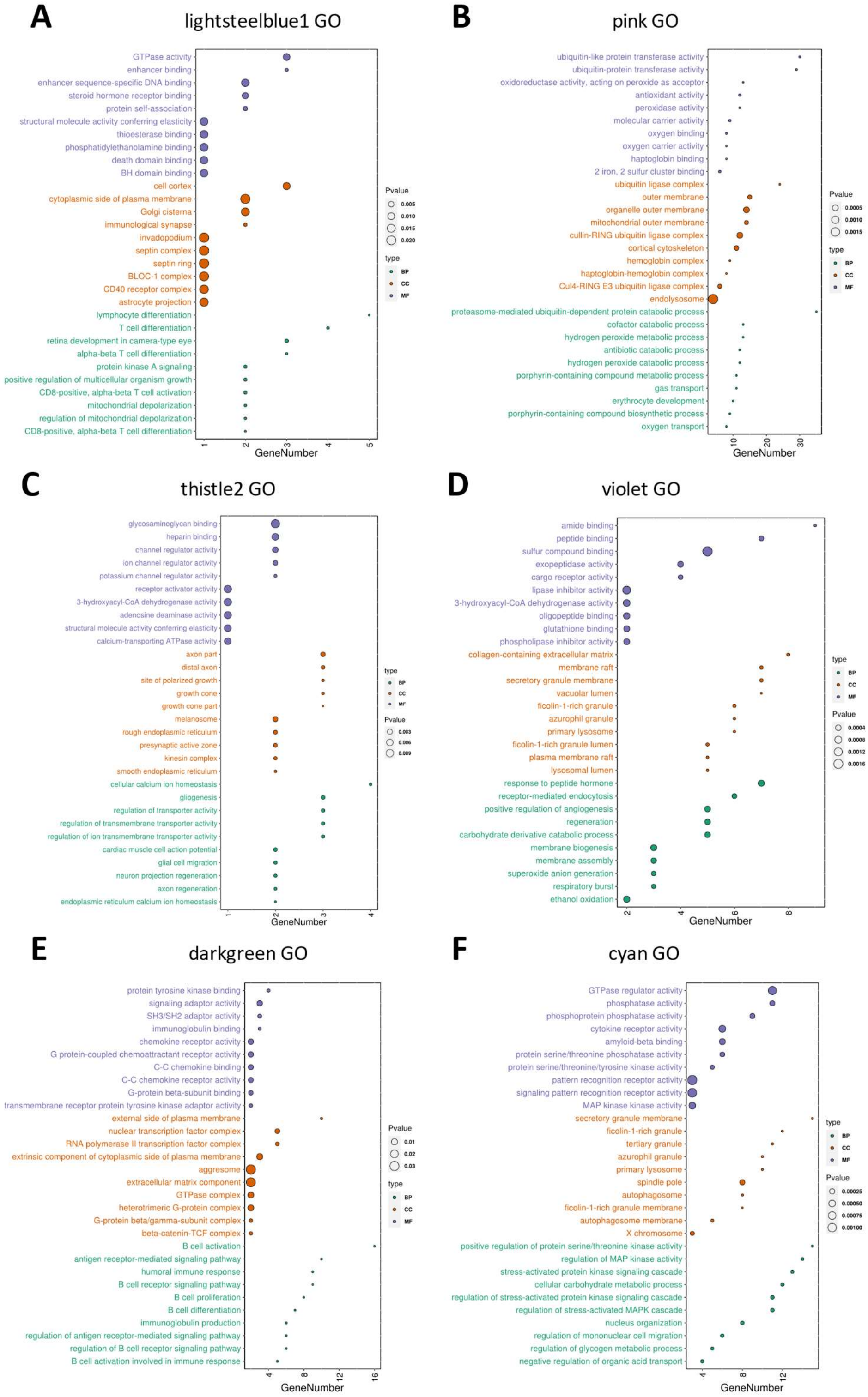

Supplement Figure S2. GO enrichment analysis in key modules

Under the threshold of $P < 0.05$ and $q < 0.2$, the top10 enrichment results of GO biological process (BP), GO molecular function (MF), GO cell component (CC) in each key module were showed (**Table S8**).

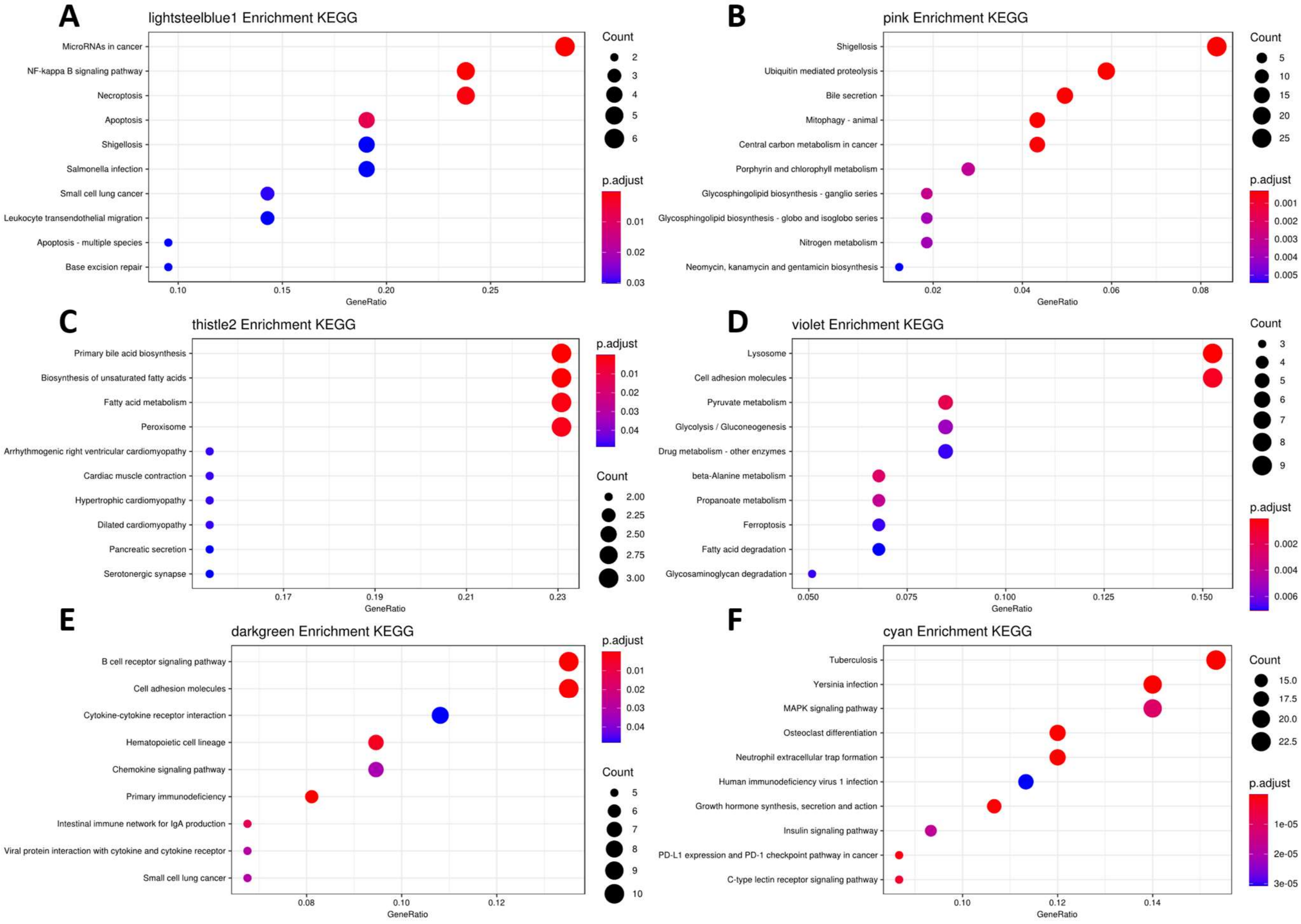

Supplement Figure S3. KEGG enrichment analysis in key modules

Under the threshold of $P < 0.05$ and $q < 0.2$, the top 10 enrichment results KEGG pathway in each key module were showed (**Table S8**).

Abbreviations: KEGG, Kyoto Encyclopedia of Genes and Genomes

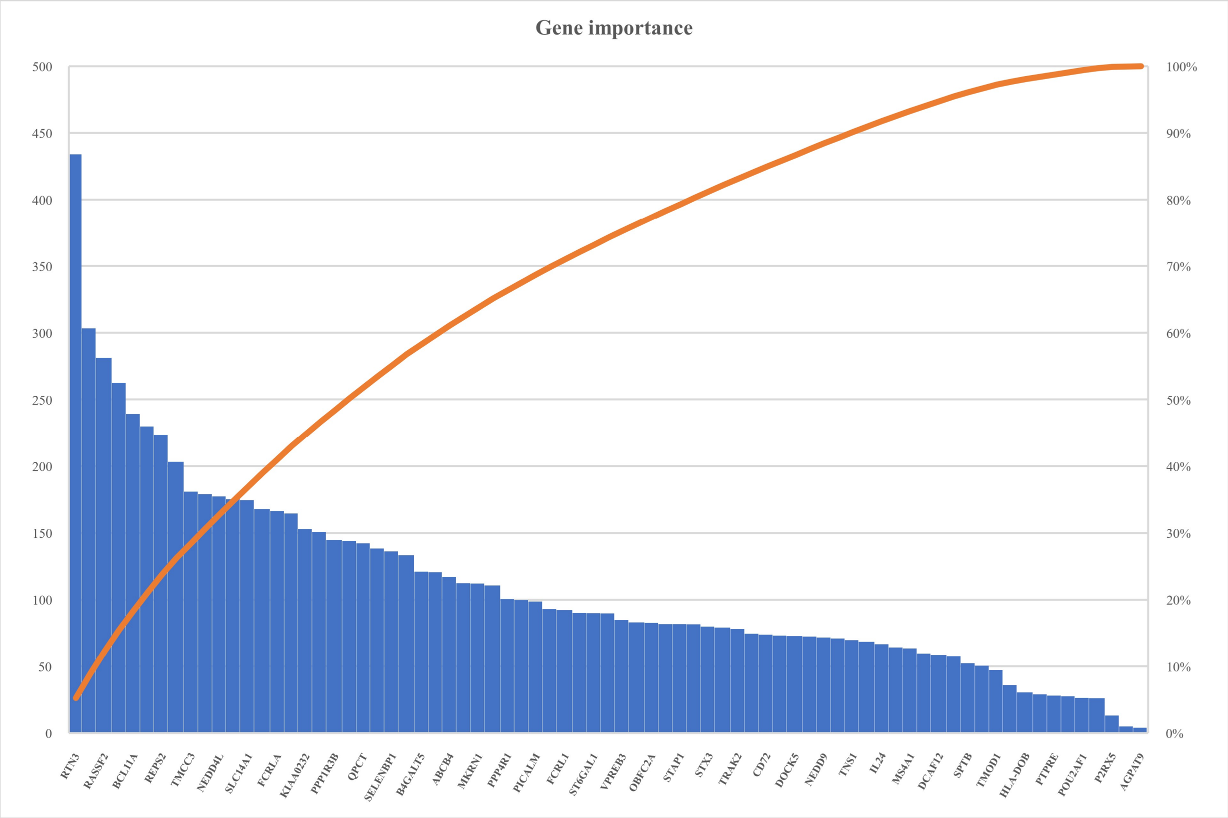

Supplement Figure S4. The overall importance ranking of hub genes

The Pareto chart shows that the overall importance ranking of 91 hub genes in seven different models. The left vertical coordinate represents the value of the gene importance, and the ordinate on the right represents the percentage of the gene importance in the population. The analysis line represents the cumulative frequency, and the abscissa represents the various factors that affect the quality, arranged from left to right according to the degree of influence. Through the analysis of the Pareto chart, the critical genes affecting the predictive ability of the model can be found. The top 10 hub genes are *RTN3, RASSF2, TCL1A, BCL11A, RANBP10, REPS2, VCAN, TMCC3, EPB41*, and *NEDD4L*, which account for approximately 30% of cumulative importance (**Table 2**).

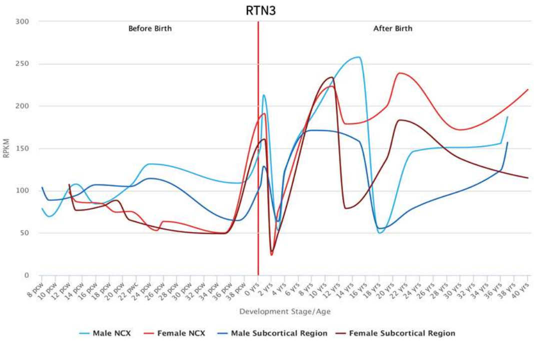
RTN3
Before Birth
After Birth
RPKM
Development Stage/Age
Male NCX
Female NCX
Male Subcortical Region
Female Subcortical Region

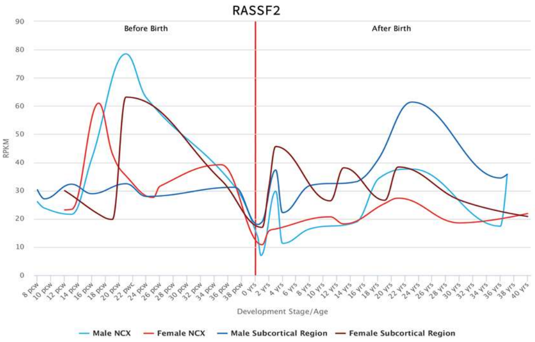
RASSF2
Before Birth
After Birth
RPKM
Development Stage/Age
Male NCX
Female NCX
Male Subcortical Region
Female Subcortical Region

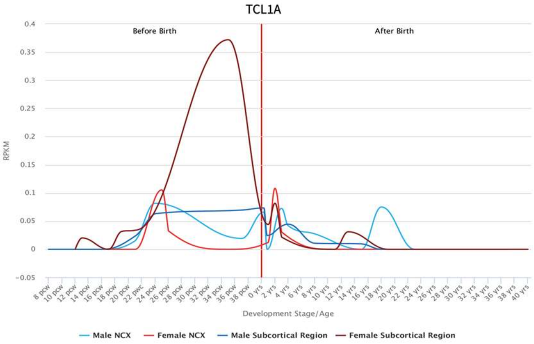
TCL1A
Before Birth
After Birth
RPKM
Development Stage/Age
Male NCX
Female NCX
Male Subcortical Region
Female Subcortical Region

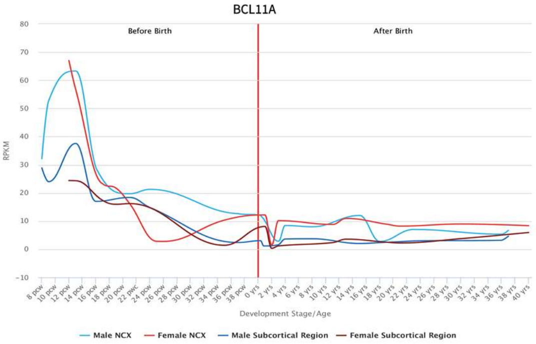
BCL11A
Before Birth
After Birth
RPKM
Development Stage/Age
Male NCX
Female NCX
Male Subcortical Region
Female Subcortical Region

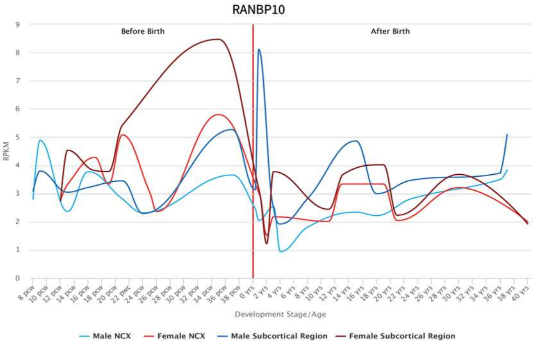
RANBP10
Before Birth
After Birth
RPKM
Development Stage/Age
Male NCX
Female NCX
Male Subcortical Region
Female Subcortical Region

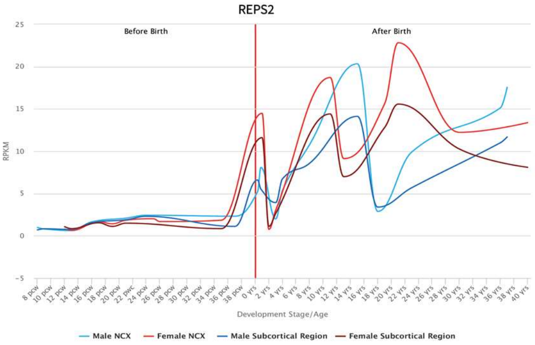
REPS2
Before Birth
After Birth
RPKM
Development Stage/Age
Male NCX
Female NCX
Male Subcortical Region
Female Subcortical Region

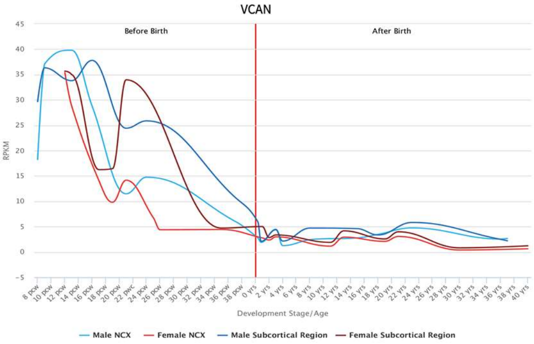
VCAN
Before Birth
After Birth
RPKM
Development Stage/Age
Male NCX
Female NCX
Male Subcortical Region
Female Subcortical Region

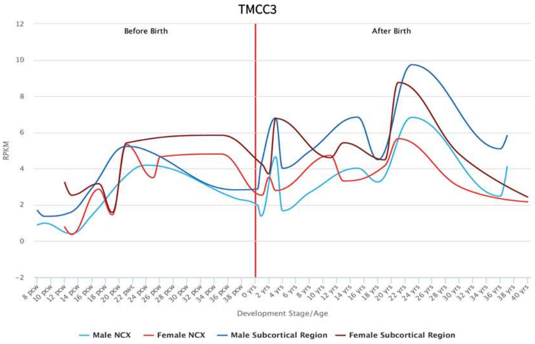
TMCC3
Before Birth
After Birth
RPKM
Development Stage/Age
Male NCX
Female NCX
Male Subcortical Region
Female Subcortical Region

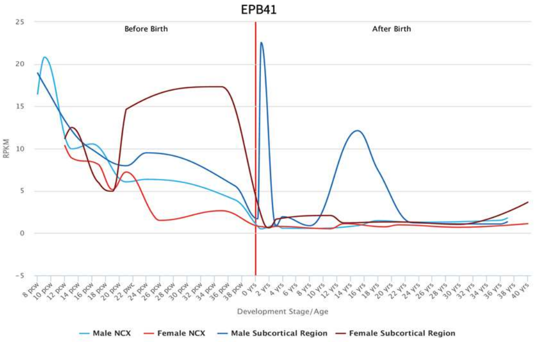
EPB41
Before Birth
After Birth
RPKM
Development Stage/Age
Male NCX
Female NCX
Male Subcortical Region
Female Subcortical Region

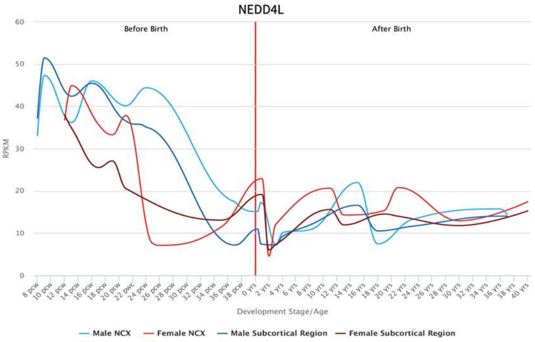
NEDD4L
Before Birth
After Birth
RPKM
Development Stage/Age
Male NCX
Female NCX
Male Subcortical Region
Female Subcortical Region

Supplement Figure S5. Transcriptional expression profiles of the top 10 hub genes in the developing human brain across genders.

GenTree (http://gentree.ioz.ac.cn/index.php) was used to display the expression levels of potential genes at various developmental stages of the human brain. The change curves of Reads Per Kilobase per Million mapped reads (RPKM) levels of top 10 hub genes from 8 post-conception week (PCW) to 40 years were calculated using RNA-sequencing data from BrainSpan (http://www.brainspan.org/). Details of the BrainSpan atlas can be found at http://help.brain-map.org/display/devhumanbrain/Documentation. The different expression patterns of males and females were compared for each gene in neocortex (NCX) and subcortical region respectively.
